# Exploring the genomic basis of early childhood caries: a pilot study

**DOI:** 10.1111/ipd.12344

**Published:** 2017-10-23

**Authors:** Jami L. Ballantine, Jenna C. Carlson, Andrea G. Ferreira Zandoná, Cary Agler, Leslie P. Zeldin, Richard Gary Rozier, Michael W. Roberts, Patricia V. Basta, Jason Luo, Mikafui E. Antonio‐Obese, Daniel W. McNeil, Robert J. Weyant, Richard J. Crout, Rebecca L. Slayton, Steven M. Levy, John R. Shaffer, Mary L. Marazita, Kari E. North, Kimon Divaris

**Affiliations:** ^1^ Department of Pediatric Dentistry School of Dentistry University of North Carolina‐Chapel Hill Chapel Hill NC USA; ^2^ Center for Craniofacial and Dental Genetics School of Dental Medicine University of Pittsburgh Pittsburgh PA USA; ^3^ Department of Biostatistics Graduate School of Public Health University of Pittsburgh Pittsburgh PA USA; ^4^ Department of Human Genetics Graduate School of Public Health University of Pittsburgh Pittsburgh PA USA; ^5^ Department of Operative Dentistry School of Dentistry University of North Carolina‐Chapel Hill Chapel Hill NC USA; ^6^ Oral and Craniofacial Health Sciences School of Dentistry University of North Carolina‐Chapel Hill Chapel Hill NC USA; ^7^ Department of Health Policy and Management Gillings School of Global Public Health University of North Carolina‐Chapel Hill Chapel Hill NC USA; ^8^ Department of Epidemiology Gillings School of Global Public Health University of North Carolina‐Chapel Hill Chapel Hill NC USA; ^9^ Biospecimen Processing Facility core University of North Carolina Chapel Hill NC USA; ^10^ Lineberger Comprehensive Cancer Center School of Medicine University of North Carolina Chapel Hill NC USA; ^11^ Mammalian Genotyping core University of North Carolina Chapel Hill NC USA; ^12^ Departments of Dental Practice & Rural Health and Psychology West Virginia University Morgantown WV USA; ^13^ Department of Dental Public Health and Information Management School of Dental Medicine University of Pittsburgh Pittsburgh PA USA; ^14^ Department of Periodontics School of Dentistry West Virginia University Morgantown WV USA; ^15^ Department of Pediatric Dentistry School of Dentistry University of Washington Seattle WA USA; ^16^ Department of Preventive and Community Dentistry University of Iowa College of Dentistry Iowa City IA USA; ^17^ Department of Epidemiology University of Iowa College of Public Health Iowa City IA USA; ^18^ Department of Oral Biology School of Dental Medicine University of Pittsburgh Pittsburgh PA USA; ^19^ Department of Psychiatry School of Medicine University of Pittsburgh Pittsburgh PA USA; ^20^ Clinical and Translational Science Institute School of Medicine University of Pittsburgh Pittsburgh PA USA

## Abstract

**Objective:**

A genetic component in early childhood caries (ECC) is theorized, but no genome‐wide investigations of ECC have been conducted. This pilot study is part of a long‐term research program aimed to: (1) determine the proportion of ECC variance attributable to the human genome and (2) identify ECC‐associated genetic loci.

**Methods:**

The study's community‐based sample comprised 212 children (mean age=39 months; range = 30–52 months; males = 55%; Hispanic/Latino = 35%, African‐American = 32%; American Academy of Pediatric Dentistry definition of ECC prevalence = 38%). Approximately 2.4 million single nucleotide polymorphisms (SNPs) were genotyped using DNA purified from saliva. A *P *<* *5 × 10^−8^ criterion was used for genome‐wide significance. SNPs with *P *<* *5 × 10^−5^ were followed‐up in three independent cohorts of 921 preschool‐age children with similar ECC prevalence.

**Results:**

SNPs with minor allele frequency ≥5% explained 52% (standard error = 54%) of ECC variance (one‐sided *P *=* *0.03). Unsurprisingly, given the pilot's small sample size, no genome‐wide significant associations were found. An intergenic locus on 4q32 (rs4690994) displayed the strongest association with ECC [*P *=* *2.3 × 10^−6^; odds ratio (OR) = 3.5; 95% confidence interval (CI) = 2.1–5.9]. Thirteen loci with suggestive associations were followed‐up – none showed evidence of association in the replication samples.

**Conclusion:**

This study's findings support a heritable component of ECC and demonstrate the feasibility of conducting genomics studies among preschool‐age children.

## Introduction

Early childhood caries (ECC) is a persistent and possibly growing public health problem. In the USA, recent data suggest that one of four children age 6 or young have experienced ECC when defined according to AAPD criteria [the presence of one or more decayed (non‐cavitated or cavitated lesions), missing (due to caries), or filled tooth surfaces in any primary tooth in a child under the age of six]^1^. Moreover, recent evidence suggests that ECC prevalence may be increasing, particularly among ethnic minority and socially disadvantaged populations, leading to substantial disparities in children's oral health^2^. Joint efforts of professional, academic, community, and policy stakeholders are focused on addressing this important health problem^3^, which tends to disproportionally affect children in families from lower socioeconomic strata^4^.

From a pathogenetic standpoint, dental caries results from complex interactions among acid‐producing members of the biofilm, fermentable carbohydrates, and many host factors, including susceptible tooth surfaces and saliva. For this reason, caries has been thought to be largely modulated by behavioural and environmental risk factors, such as diet and fluoride exposure. The disease is associated with substantial functional, quality of life, and economic costs, whereas restorative care is not curative and often fails to arrest the disease process^5^. Since the late 1950s, dental caries has been shown to have a substantial genetic component^6^, ^7^, ^8^. Estimates of the disease variance proportion explained by genetics (often referred to as “heritability”) have ranged from 30% to 70%, with higher estimates found for primary versus permanent dentition caries^9^, ^10^, ^11^, ^12^. Numerous candidate‐gene studies have since been conducted to investigate the postulated role of several hypothesized genes in caries aetiology in children and adults^13^. Studies in this body of literature have largely targeted enamel development and mineralization genes, as well as genes involved in the immune response in early childhood. As reviewed by Vieira and colleagues^12^, these studies have had mixed results and currently no consensus knowledge of the genetic basis of ECC exists. This is not surprising, given the very small sample size of most dental caries investigations (typically up to few hundred subjects) compared to genomics studies conducted for other common diseases and traits, frequently including upwards of 50,000–100,000 participants.

Genome‐wide association studies (GWAS) have been successful in identifying associations between common genetic variants [primarily single nucleotide polymorphisms (SNPs)] and several common diseases or traits, including asthma, diabetes, colorectal cancer, cardiovascular, and psychiatric conditions^14^. The advent of GWAS has enabled the ‘unbiased’ scan (i.e., a hypothesis‐free exploration) of millions of SNPs across the human genome, in an efficient manner. It is anticipated that GWAS will illuminate the contribution of genomics in oral health and care^15^, although progress to date has been slow. Only one GWAS of caries in the primary dentition has been conducted, using a sample of 1300 European‐American (white) children aged 3–12 years old^16^. This study identified several genetic loci with suggestive evidence of association that could have plausible biological roles in childhood caries, but found no genome‐wide statistically significant associations. To address the knowledge gap of genomics in ECC, we have embarked on a long‐term research programme aimed to study the genetic underpinnings of ECC among a large sample of community‐based preschool‐age children. Here, we present the results of a pilot GWAS of ECC (i.e., among children aged 71 months old or younger), conducted in a multi‐racial/ethnic sample of preschool‐age children enrolled in a community‐based study of childhood oral health. As part of this pilot and feasibility study, we estimated the heritability of ECC and attempted to replicate loci with suggestive associations in the discovery sample in three external cohorts of preschool‐age children.

## Materials and methods

This pilot GWAS was conducted using DNA extracted from saliva samples collected from a multi‐ethnic sample (Table 1) of 212 low‐income preschool children (ages 2–4) enrolled in the Zero‐Out Early Childhood Caries (ZOE) study (UNC‐Chapel Hill IRB #08‐1185) previously reported by Barakat *et al*.^17^ The planned recruitment for a large‐scale GWAS of ECC is approximately 6000 children enrolled in Head Start centres across North Carolina, currently undertaken as the ZOE 2.0 study. Detailed enrolment procedures, inclusion and exclusion criteria of the ZOE study, are reported in Born *et al*.^18^ Briefly, participating children were enrolled in Early Head Start programs or living in nearby ‘control’ locations in North Carolina and were examined by a single clinical examiner at the child's preschool or a nearby community location using portable dental equipment. According to the AAPD ECC definition, any child with a single decayed (cavitated or non‐cavitated), missing (presumably due to caries), or filled tooth surface was classified as having ECC. A secondary measure, caries severity, was ascertained using the dmfs index, which is the sum of surface‐level diagnoses for decayed, missing‐extracted due to caries or restored tooth surfaces. Diagnosis of surface‐level caries lesions was based on NIDCR visual criteria at the non‐cavitated level (i.e., d_1_) without radiographs following a toothbrush prophylaxis, compressed air‐drying and artificial light with the use of magnification. Excellent intra‐examiner reliability was achieved for surface‐level caries lesion diagnoses: *κ* = 0.85, 95% CI = 0.83–0.88, upon duplicate examination of 23 children within a 3‐day period. Sociodemographic and behavioural risk factors were collected via structured, computer‐assisted parent interviews that were administered in English or Spanish.

**Table 1:** Demographic characteristics of the 212 preschool‐age children participating in the ZOE GWAS, overall, and by early childhood caries (ECC) status

	All	ECC	Caries‐free
	*n* (col. %)	*n* (row. %)	*n* (row %)
Entire sample	212 (100)	78 (38)	132 (62)
Sex			
Male	116 (55)	50 (43)	66 (57)
Female	96 (45)	30 (31)	66 (69)
Race/ethnicity			
African American	67 (32)	26 (39)	41 (61)
Hispanic American/Latino	74 (35)	34 (46)	40 (54)
European American	49 (23)	13 (27)	36 (73)
Native American	21 (10)	6 (29)	15 (71)
Other	1 (0)	1 (100)	0 (0)

Saliva samples were collected alongside the clinical examinations using the Oragene DNA Genotek OG‐575 kit. Consent for saliva donation for genomics analyses was given by 96% (*n* = 331) of eligible children in the pilot study, and saliva samples were obtained from 64% of those (*n* = 213). The most frequent reasons for not obtaining a saliva sample were lack of cooperation (18%) and inadequate salivation (12%; Fig. S1). DNA extraction, quantitation, and quality assessment were performed at the UNC‐Chapel Hill Biospecimen Processing facility with good results, that is, sufficient quantity and quality DNA was obtained for high‐density genotyping purposes. Mean DNA yields according to quantitation method were [*μ*g (SD)]: optical density (OD) – 44.1 (26.7), Picogreen (PG) – 29.1 (15.6), human‐specific RNAseP – 3.9 (1.6; Fig. S2). Moreover, >80% of samples had A260/A280 ratio between 1.6–2.0 and 260/230 ratio above 1.5.

Genotyping was performed at the UNC‐Chapel Hill Mammalian Genotyping core using the Illumina HumanOmni2.5‐8 bead chip (offering ~2.4million markers). Genotyping quality control procedures included HapMap‐CEPH trios and duplicates, seven blind duplicate samples, identification of sex and sample mismatches, and generation of sample call and error rates. After the exclusion of one contaminated sample, 212 high‐quality genotyped samples were obtained [i.e., no sex mismatches, median (range) – sample call rate = 99.8% (96.1%–99.9%) and concordance rate = 99.996% (99.991%–99.997%)] and were carried forward to analyses.

After quality control, ~2.3 million SNPs were used to estimate heritability of ECC, both with and without adjustment for age, sex, and ancestry using Genome‐wide Complex Trait Analysis (GCTA) software^19^ and various Minor Allele Frequency (MAF) thresholds of 1%, 5%, and 10%. Low‐frequency (MAF 0.5%–5%) and rare (MAF <0.5%) polymorphisms can contribute to variability in complex traits or disease; however, due to the small sample size of this pilot GWAS analysis and the likelihood of inducing spurious findings, they were excluded for analyses and reporting. To estimate and test heritability, GCTA employs a random‐effects mixed linear model and restricted maximum‐likelihood regression adjusting for age, sex, and ancestry^19^. Heritability was estimated for the binary ECC case definition and the continuous measure of disease severity, the conventional d_1,2‐3_mfs index (the sum of decayed, missing due to caries, and filled/restored primary tooth surfaces) combining non‐cavitated and cavitated caries lesions. Of note, *P*‐values reported for heritability estimates are one‐sided, as variance explained (by genetics or any other source) cannot take negative values.

Genetic associations of the ~1.4 million common SNPs (MAF ≥5%) with the binary ECC case definition were tested using logistic regression while adjusting for age, sex, and ancestry. Ancestry adjustments were performed to control for population stratification^20^ (i.e., systematic differences in allele frequency between cases and controls that can induce spurious associations) via the generation of 10 ancestry principal components (PCs). Although these PCs do not have a straightforward interpretation, they represent axes of common, genetically determined ancestry in the study sample, and are treated as covariates (e.g., confounders) in statistical analyses. The conventional genome‐wide significance threshold for GWAS is *P *<* *5 × 10^−8^. In addition, a more lenient (*P *<* *10^−5^) threshold to identify ‘suggestive’ evidence of association, albeit non‐significant, was considered as a means of highlighting additional candidate genes. Loci of interest were visualized using LocusZoom software^21^.

Association of the prioritized SNPs (*P *<* *5 × 10^−5^) was examined in three independent cohorts comprising 921 preschool children from the Center for Oral Health Research in Appalachia study^22^ (COHRA, *n* = 326; mean age = 35 months; ECC prevalence = 25%), Iowa Fluoride Study^23^ (IFS) *n* = 348; mean age = 60 months; ECC prevalence = 35%) and the Genetic, Environment and Health Initiative Research Study^24^, (GEIRS, *n* = 247; mean age = 48 months; ECC prevalence = 25%). Replication was considered using three criteria: (1) consistency in the direction of association (i.e., the same risk allele observed across samples) and *P*‐values less than the Bonferroni‐corrected statistical significance threshold in all three replication samples; (2) directional consistency and *P*‐values less than a nominal threshold (*P *<* *0.05); and (3) directional consistency between prioritized SNPs associations in the discovery (ZOE) and the three replication samples, as determined by a binomial test (*P *<* *0.05).

## Results

The prevalence of ECC among the 212 participating children (mean age = 39 months; range = 30–52 months) was 38%. The demographic characteristics of this multi‐ethnic/racial sample are provided in Table 1. When considering all common SNPs (MAF ≥5%; approximately 1.4 million), the heritability (*h* ^2^) of ECC was 52%, *P *=* *0.03 (or *h* ^2^ = 44%, when including all SNPs with MAF ≥1%, that is, ~1.9 million SNPs). This estimate diminished after adjustment for ancestry: *h* ^2^ = 13% (*P *=* *0.4). This lower proportion corresponds to the ECC variance explained by genetics that is common and shared across all ancestry (and effectively racial/ethnic) groups. Similarly, heritability was markedly lower, at 14% (*P *=* *0.01) for ECC severity (d_1‐2,3_mfs index) using the same set of common SNPs (MAF ≥ 5%) compared to the binary ECC case definition (Table 2).

**Table 2:** Phenotypic variance explained for ECC case status and severity (d_1‐2,3_mfs index) among the 212 preschool‐age children enrolled in the ZOE GWAS

	ECC case status (binary definition)		ECC severity (d_1_d_2,3_mfs index)	
	Variance explained (SE)	LR *P*‐value	Variance explained (SE)	LR *P*‐value
All SNPs (*n* = 2,331,188)				
Only SNPs considered	0.43 (0.36)	0.043	0.06 (0.08)	0.12
+Age, sex	0.38 (0.36)	0.07	0.06 (0.08)	0.13
MAF >0.01 (*n* = 1,877,037)				
Only SNPs considered	0.44 (0.39)	0.034	0.07 (0.08)	0.08
+Age, sex	0.39 (0.39)	0.061	0.07 (0.08)	0.08
MAF ≥0.05 (*n* = 1,382,931)				
Only SNPs considered	0.52 (0.55)	0.026	0.14 (0.14)	0.01
+Age, sex	0.43 (0.50)	0.050	0.13 (0.14)	0.02
MAF ≥0.10 (*n* = 986,805)				
Only SNPs considered	0.48 (0.53)	0.026	0.21 (0.20)	0.006
+Age, Sex	0.39 (0.48)	0.050	0.19 (0.19)	0.008

LR, likelihood ratio χ^2^ test – one‐sided *P*‐value; SE, standard error; MAF, minor allele frequency.

**Figure 1:**
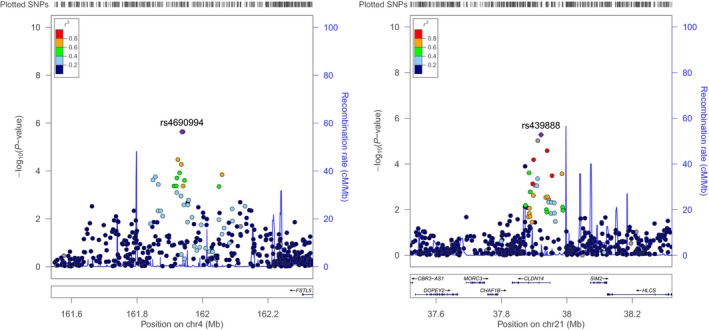
Regional association plots of the top two loci that emerged with the strongest evidence of association (lowest *P‐*values, even though not genome‐wide significant) with ECC among the 212 preschool‐age children participating in the ZOE genome‐wide association study, left panel (a): 4q32 locus (lead SNP: rs4690994, *P* = 2.3 × 10^−6^; odds ratio (OR) = 3.5; 95% confidence interval (CI) = 2.1–5.9); right panel (b): the 20q22 locus (lead SNP rs439888; *P* = 5.3 × 10^−6^; rs439888 intronic SNP; OR = 3.6; 95% CI = 2.1–6.2). Position on the *x*‐axis corresponds to genomic coordinates (position), and the position on the *y*‐axis corresponds to each SNP's –log_10_(*P*‐value). The top, or ‘lead’, SNP is coloured purple, whereas other polymorphisms are colour coded by their *r* ^2^, a measure of linkage disequilibrium, with the lead SNP. Plots were generated using Locus Zoom^21^.

We identified 13 genetic loci demonstrating suggestive evidence of association with ECC status, with no evidence of genomic inflation (Fig. S3). Of these 13 loci, the two most significant regions were observed on 4q32 and 20q22 marked by SNPs rs4690994 and rs439888, respectively (Fig. S4). Specifically, the intergenic locus on 4q32 showed the strongest association with ECC [Fig. 1a; rs4690994; *P *=* *2.3 × 10^−6^; odds ratio (OR) = 3.5; 95% confidence interval (CI) = 2.1–5.9], followed by the 20q22 locus (Fig. 1b; rs439888 intronic to the *CLDN14* gene; *P *=* *5.3 × 10^−6^; OR = 3.6; 95% CI = 2.1–6.2). The functional role of 4q32 is currently unknown, but the *CLDN14* gene encodes a transmembrane, tight junction protein. Defects in this gene result in autosomal recessive, non‐syndromic, sensorineural deafness. Other variants in this gene have been associated with nephrolithiasis and reduced bone density.

Replication was attempted for all 13 SNPs demonstrating suggestive statistical association with ECC separately within each of the three replication samples. None of the SNPs showed genetic association in replication samples, and only 15 of the 39 SNP look‐ups showed directional association concordance.

## Discussion

This report presents the results of the first GWAS of ECC, which was conducted among a small pilot sample of 212 community‐based preschool‐age children participating in a dental public health study. First and foremost, the study demonstrates that genomics investigations of common oral health traits, including ECC, are feasible among preschool‐age children in non‐clinical settings – with a key enabling feature being saliva collection, which allowed the extraction of high‐quality DNA suitable for high‐density genotyping. Second, the results support the existence of a substantial genetic component for ECC, which lends itself to future larger, comprehensive genomics investigations. Finally, although this pilot study did not discover any ECC‐associated genetic markers, it highlighted several loci that could be of potential relevance to dental caries and worth further investigation in future, larger association studies.

Insights from genomics studies of ECC can aid in the discovery of previously unknown or unsuspected mechanistic pathways and inform risk assessment^25^; a detailed account of individual susceptibility, behaviours, and environment can help the tailoring or optimization of prevention and treatment, and thus serve as the basis of precision medicine or precision dentistry. In this pilot study, there were no statistically confirmed genome‐wide association results and none of the suggestive findings were replicated in the three independent samples of young children. This could be due to several reasons: (1) the genetic loci reported here have no real influence on ECC; in other words, they reflect a ‘winner's curse’ phenomenon^26^, (2) the very low statistical power of the discovery GWAS, (3) the equally small sample of the individual replication cohorts and the resulting low statistical power, (4) unobserved or unmeasured heterogeneity between the studied populations, and (5) outcome misclassification [i.e., the ECC definition including cases that range from ‘marginal’ (e.g., one affected surface) to rampant, as well as both cavitated and non‐cavitated, or early stage caries lesions]. Although no genetic loci met the significance threshold, it is likely that larger, future studies will be successful in discovering specific genetic influences of the disease.

Despite these limitations, our findings demonstrate the feasibility of the overall approach, from the conduct of clinical examinations of young children under field conditions to the genotyping of millions of genetic markers using saliva samples obtained during those exams. Of note, our approach utilizing commercial saliva sampling kits for DNA extraction enabled this procedure to be conducted in remote locations without the need for specialized equipment: Saliva samples are stable and can be stored at room temperature for up to 5 years until DNA extraction takes place in a laboratory setting. In this study, all saliva samples obtained yielded high‐quality genotype data, with the exception of only one sample that was contaminated and was discarded from all analyses. We were able to obtain saliva samples from approximately two‐thirds of the participating children and determined that non‐cooperation and inadequate salivation were the most frequent reasons for not being able to obtain a saliva sample – this is not surprising because the children were very young (2‐ and 3‐year‐olds). In our experience, means for increasing the likelihood of obtaining usable saliva samples in this very young age group include obtaining the sample at the beginning of the visit and 30–60′ after they have had a meal or drink, employing examiners who are trained in the management of very young children, and talking about or presenting photographs of food or sweet snacks during the sample collection.

Although this pilot study did not find any (and was underpowered for the discovery of) new genetic loci for ECC, it suggests that a sizeable heritable component of ECC exists. Our estimates of heritability are in general agreement with previous reports in the literature^12^. It is noteworthy that the percentage of ECC variance explained ranged between 44% and 52% in our GWAS, with little variation attributable to different sets (still, millions) of SNPs used. Surface‐level clinical diagnoses were made, uniformly, without radiographs; a feature that likely led to underestimation of caries experience. Of note, heritability was markedly lower for the dmfs index compared to the ECC case definition. This finding was somewhat expected because variations introduced by restorative dental treatment (e.g., placement of a full‐coverage, stainless steel crown, coded as a 5‐surface restoration versus a pre‐existing 2‐surface caries lesion) can inflate the underlying clinical caries experience. An alternative approach to circumvent this issue could be the interrogation of the diseased‐only surfaces rather than the complete dmfs index; however, this metric could also be confounded by access to care issues, which would affect the ratio of treated vs. untreated disease. On the other hand, heritability was substantially lower when our models were adjusted for ancestry via the inclusion of 10 principal components; this result should be treated with caution, as such adjustments can produce statistically unstable estimates due to the small sample size. Nevertheless, it is indicative of the impact of race/ethnicity‐specific influences, which are at play in a racially mixed sample like in our study.

Our study did not consider traditional risk factors for ECC, including socioeconomic status, diet, oral hygiene, and fluoride exposures. This could be performed via the conduct of stratified analyses or via the inclusion of additional terms for these factors in genetic models. As noted earlier, traditional risk factors, although strongly associated with the trait or disease under study, are not confounders of the genetic associations and adjustments for these factors are not performed^27^. Nevertheless, stratification by such factors or examination of gene–environment (e.g., fluoride) interactions have been informative in previous investigations^16^, ^28^ and should be explored in cases where the sample size permits. Interestingly, some biological pathways that are genetically controlled^16^ could be operating via clinical^29^ (e.g., saliva composition, enamel properties, immunity, and metabolism) or behavioural risk factors, with sweet taste preference being suggested by relatively recent studies^30^.

In sum, the major novelty and strength of this study was the opportunity to do an unbiased scan of the human genome without *a priori* hypotheses for the first time, in a narrow‐age range sample, appropriate for the study of ECC. This study also benefits from the uniform clinical examination protocol and the opportunity to replicate or generalize its findings to external samples of almost one thousand preschool‐age children. Lastly, race‐ or ethnicity‐specific results were not examined in these analyses due to the small sample size of the respective strata; although this study characteristic could further reduce the statistical power, we consider that the inclusion of under‐studied racial/ethnic groups in this pilot investigation is a novel, positive element.

**Why this paper is important to paediatric dentists**

- The study confirms that a substantial heritable component of ECC exists.
- Genomics studies are feasible among preschool‐age children using saliva samples obtained during dental examinations‐‐good quality and sufficient amount of DNA can be obtained from saliva, suitable for high‐density genotyping.
- Future, large or collaborative multi‐ethnic/multi‐racial studies, are likely to identify specific genetic influences for ECC, which can help better understand, prevent and treat this early‐onset, aggressive childhood disease.

## Author contributions

R.L.S., S.M.L, J.R.S., M.L.M, and K.D. conceived the research ideas in each respective cohort; A.F.Z., L.P.Z, R.G.R., M.E.A, R.L.S., and K.D. designed the studies and collected the data; J.LB., J.C.C., C.A, and K.D. conducted data analyses; P.V.B., R.L.S., and J.L conducted the laboratory analyses; J.R.S., M.W.R., D.W.M, and R.J.C contributed to the interpretation of the results and critically revised the manuscript; J.L.B. and K.D. led the writing; all authors reviewed, contributed to and approved the final version of the manuscript.

## Conflict of interest

The authors declare no conflict of interest with respect to this work.

## Supporting information


**Fig. S1.** Results of the saliva sample collection process among the 346 preschool‐age children participating in the ZOE study.
**Fig. S2.** Quantitation of DNA purified from saliva samples among the 213 preschool‐age children that donated a sample for the ZOE GWAS study.
**Fig. S3.** Quantile‐Quantile (QQ) plot of GWAS results of ECC among the 212 preschool‐age children participating in the ZOE GWAS.
**Fig. S4.** Manhattan plot of the ~1.4 million association results [y‐axis corresponds to ‐log_10_(p‐value)] of genotyped SNPs with the ECC case definition, arranged by chromosome, among the 212 preschool‐age children participating in the ZOE GWAS.
