# Supplementary material for: Exploring the genomic basis of early childhood caries: a pilot study
Source: Int J Paediatr Dent. 2017 Oct 23;28(2):217–25. doi: 10.1111/ipd.12344 (PMC5811369; doi:10.1111/ipd.12344)

**Supplemental Figure S1**. Results of the saliva sample collection process among the 346 preschool-age children participating in the ZOE study. Consent for saliva donation was given by 96% (n=331) of children’s parents/caregivers and samples were successfully obtained from 64% of these children (n=213). Uncooperative behavior was the most common (18%) reason for non-collection, followed by inadequate salivation (12%).

**Supplemental Figure S2**. Quantitation of DNA purified from saliva samples among the 213 preschool-age children that donated a sample for the ZOE GWAS study. Sufficient DNA quantities for genotyping were obtained, with mean yields [μg (SD)]: Optical Density—8.6 (5.2), Picogreen—5.7 (3.0), human-specific RNAseP assay—3.9 (1.6). Forty-eight percent of samples had A260/A280 ratio between 1.7-1.9.

**Supplemental Figure S3**. Quantile-Quantile (QQ) plot of GWAS results of ECC among the 212 preschool-age children participating in the ZOE GWAS. This QQ plot illustrates ~1.4 million observed (y-axis) versus expected (x-axis) association results [-log_10_(p-values)] based on logistic regression genetic models of ECC using common (MAF ≥0.05) SNPs. The models assumed multiplicative allelic effects and included adjustment for ancestry (10 principal components), age and sex.

.


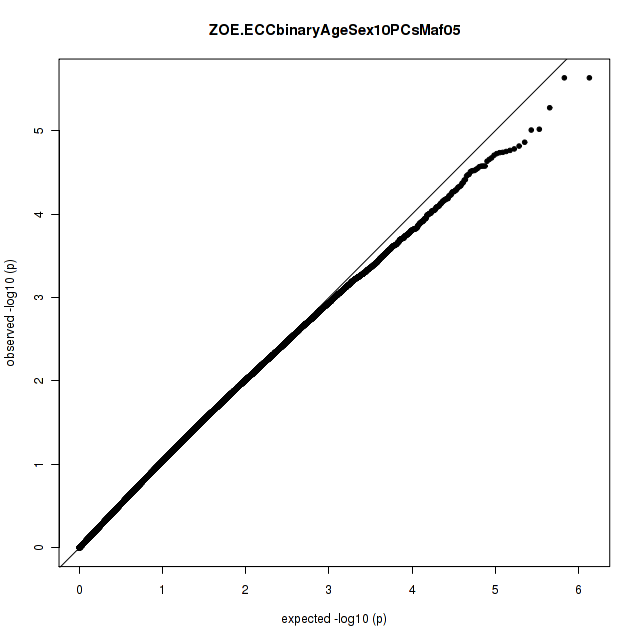


**Supplemental Figure S4**. Manhattan plot of the ~1.4 million association results [y-axis corresponds to -log_10_(p-value)] of genotyped SNPs with the ECC case definition, arranged by chromosome, among the 212 preschool-age children participating in the ZOE GWAS.


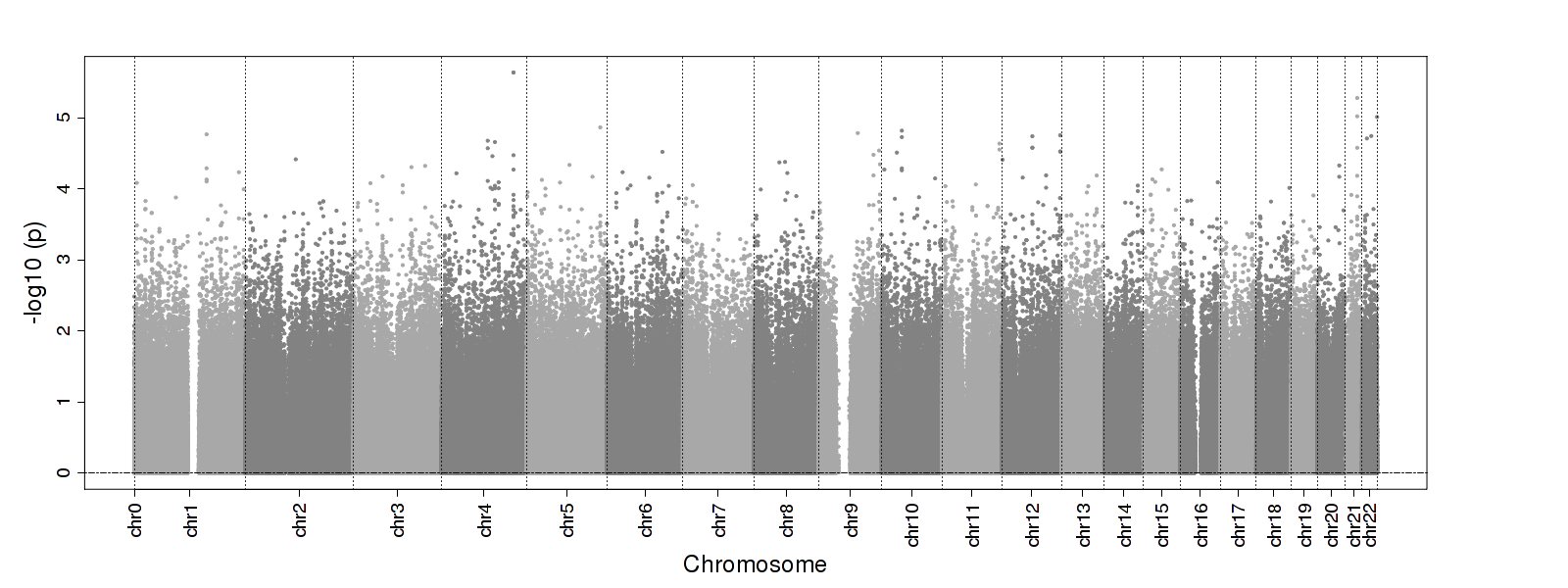

Supplement: Supplementary file 1 — Fig. S1. Results of the saliva sample collection process among the 346 preschool‐age children participating in the ZOE study. Fig. S2. Quantitation of DNA purified from saliva samples among the 213 preschool‐age children that donated a sample for the ZOE GWAS study. Fig. S3. Quantile‐Quantile (QQ) plot of GWAS results of ECC among the 212 preschool‐age children participating in the ZOE GWAS. Fig. S4. Manhattan plot of the ~1.4 million association results [y‐axis corresponds to ‐log10(p‐value)] of genotyped SNPs with the ECC case definition, arranged by chromosome, among the 212 preschool‐age children participating in the ZOE GWAS. [file IPD-28-217-s001.docx]
